# Induction and Suppression of Innate Antiviral Responses by Hepatitis A Virus

**DOI:** 10.3389/fmicb.2018.01865

**Published:** 2018-08-17

**Authors:** Xin Cao, Yu-jia Xue, Jiang-long Du, Qiang Xu, Xue-cai Yang, Yan Zeng, Bo-bo Wang, Hai-zhen Wang, Jing Liu, Kui-zheng Cai, Zhong-ren Ma

**Affiliations:** ^1^College of Life Science and Engineering, Northwest Minzu University, Engineering & Technology Research Center for Animal Cell, Lanzhou, China; ^2^Key Laboratory of Bioengineering & Biotechnology of State Ethnic Affairs Commission, Lanzhou, China; ^3^State Key Laboratory of Veterinary Etiological Biology, Lanzhou Veterinary Research Institute, Chinese Academy of Agricultural Sciences, Lanzhou, China; ^4^Hebi Precision Medical Research Institute, People's Hospital of Hebi, Hebi, China; ^5^Department of Medical Oncology People's Hospital of Hebi, Hebi, China

**Keywords:** HAV, MAVS, TRIF, NEMO, type 1 interferon

## Abstract

Hepatitis A virus (HAV) belongs to the family *Picornaviridae*. It is the pathogen of acute viral hepatitis caused by fecal-oral transmission. RNA viruses are sensed by pathogen-associated pattern recognition receptors (PRRs) such as Toll-like receptor 3 (TLR3), retinoic acid-inducible gene I (RIG-I), and melanoma differentiation-associated gene 5 (MDA5). PRR activation leads to production of type 1 interferon (IFN-α/β), serving as the first line of defense against viruses. However, HAV has developed various strategies to compromise the innate immune system and promote viral propagation within the host cells. The long coevolution of HAV in hosts has prompted the development of effective immune antagonism strategies that actively fight against host antiviral responses. Proteases encoded by HAV can cleave the mitochondrial antiviral signaling protein (MAVS, also known as IPS-1, VISA, or Cardif), TIR domain- containing adaptor inducing IFN-β (TRIF, also known as TICAM-1) and nuclear factor-κB (NF-κB) essential modulator (NEMO), which are key adaptor proteins in RIG-I-like receptor (RLR), TLR3 and NF-κB signaling, respectively. In this mini-review, we summarize all the recent progress on the interaction between HAV and the host, especially focusing on how HAV abrogates the antiviral effects of the innate immune system.

## Introduction

HAV is a positive-strand RNA virus lacking a lipid envelope. As a member of the *Picornaviridae* family, it has strong tropism for the human hepatocyte like hepatitis C virus (HCV). It is the sole member of the genus Hepatovirus. Enveloped HAV (eHAV) were formed by hijacking cellular membranes, thereby virion were protected from antibody-mediated neutralization (Feng et al., 2013). The HAV genome is ~7,500 nucleotides in length and contains a 5′-untranslated region (UTR), a single open reading frame (ORF) and a 3′-UTR with a polyadenosine tail. The large single open-reading frame encodes a polyprotein that consists of the structural genes (VP1 to VP4, i.e., P1 segment) and the non-structural genes (2A−2C, i.e., P2 segment and 3A−3D, i.e., P3 segment) (Martin and Lemon, 2006; Debing et al., 2014). The investigators use high-resolution X-ray structures to observe HAV mature virus and empty particle. The structures of the two particles are very similar, both forming an icosahedral protein capsid. However, the HAV mature virus has the small viral protein VP4, whereas the empty particle contains only the uncleaved precursor, VP0 (Wang et al., 2015b). A putative receptor for HAV, the HAV cellular receptor (HAVcr-1), is an integral membrane mucin-like glycoprotein with unknown natural function and was identified in African green monkey kidney cells (AGMK) (Kaplan et al., 1996). The human homolog (HuHAVcr-1), otherwise known as T-cell immunoglobulin and mucin-containing domain protein 1 (Tim-1), was also identified and characterized as a human HAV receptor (Feigelstock et al., 1998). In addition, Tim-3 and HAV-specific immunoglobulin A (IgA)-HAVcr-1 association facilitated virus entry into target cells (Dotzauer et al., 2000; Sui et al., 2006; Tami et al., 2007).

In this review, we summarize recent advances in the understanding of induction and suppression of innate antiviral responses by the hepatitis A virus.

## Polyprotein processing of HAV

The large ORF encodes a single ~2230 amino acid polyprotein that is cleaved into 10 mature proteins primarily by a single virally encoded proteinase, 3C^pro^ and unknown cellular protease (Schultheiss et al., 1994; Gosert et al., 1996; Debing et al., 2014). Six non-structural proteins, each involved in replication of the HAV RNA, are derived from the P2 and P3 segments of the polyprotein: 2B, 2C, 3A, 3B, 3C^pro^, and 3D^pol^. Interestingly, 3A is targeted to mitochondrial membranes (Yang et al., 2007). 3C^pro^ is a cysteine protease, while 3D^pol^ is the viral RNA-dependent RNA polymerase and the catalytic core of the replicase complex (McKnight and Lemon, 2018). The 3ABC processing intermediate is unique among picornaviruses in that it is relatively stable and has distinct activities in particle assembly (Probst et al., 1998). Processing at the 3CD site is more efficient than at 3AB and 3BC, and 3CD is proteolytically active like 3ABC (Probst et al., 1998).

## Induction of innate antiviral responses by HAV

**Figure 1:**
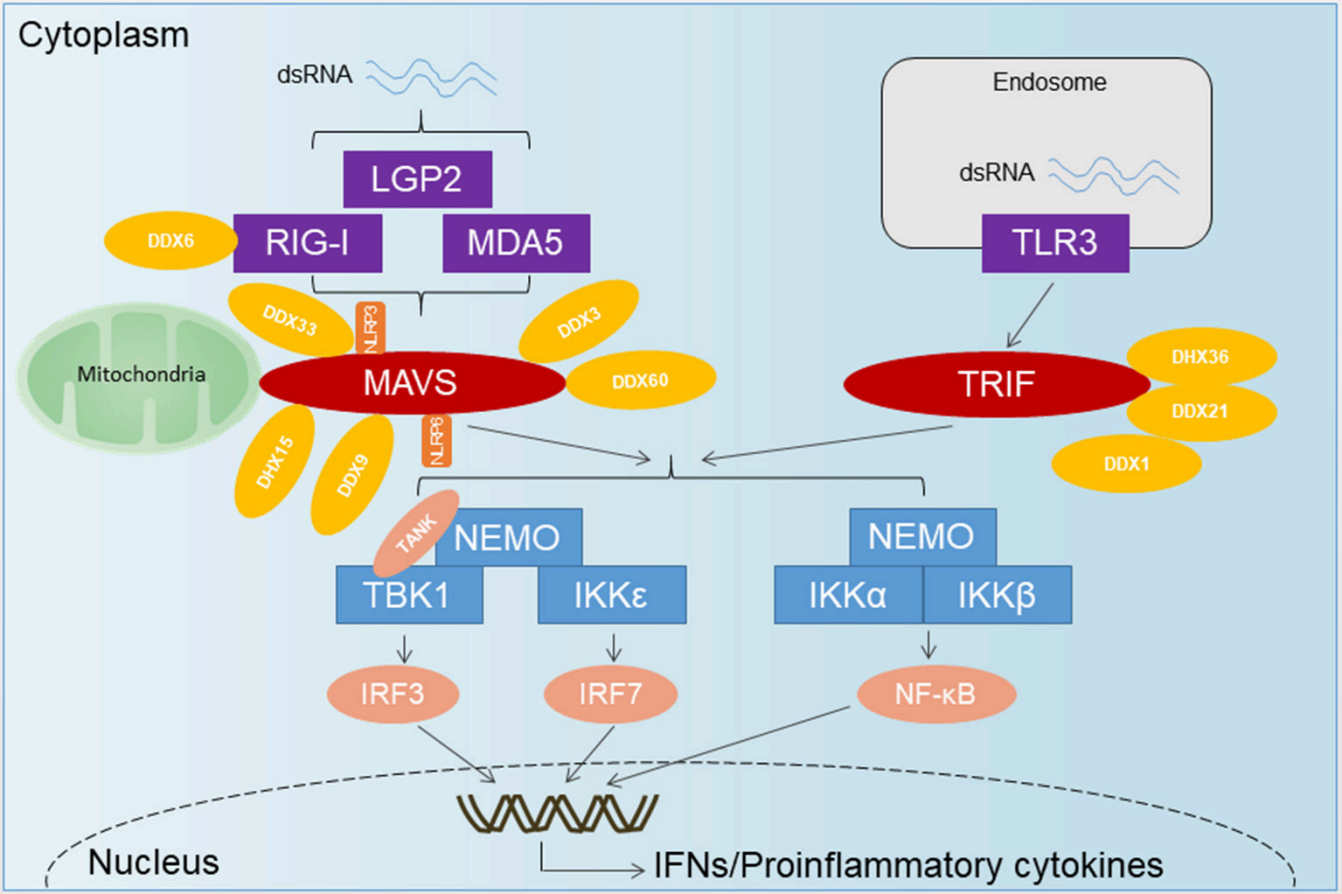
The overview of RNA-sensing pathway. TLR3 senses dsRNA and utilizes the adaptor TRIF to activate IRF3 and NF-κB. Upon recognition of dsRNA, RLRs are recruited by the adaptor MAVS located on the outer membrane of the mitochondria, leading to the activation of several transcription factors including IRF3, IRF7, and NF-κB. DExD/H-Box helicases other than the RLRs and some RNA-binding proteins have emerged as important for innate immune signaling and control of virus infection. DDX1-DDX21-DHX36 forms a complex with TRIF. DDX3 has been shown to associate with RIG-I, MDA5, and MAVS. DDX60 has been shown to bind RIG-I, whereas DHX9 has been shown to interact with MAVS. DHX33 has also been shown to bind MAVS, as well as the NLR NLRP3, to induce inflammasome assembly. DHX15 may serve as a sensor for viral RNA in the cytosol to signal NLRP6-mediated interferon responses in a MAVS-dependent manner, independent of inflammasome formation.

Hepatocytes, the primary cell type targeted by HAV for infection, express both retinoic acid activated gene I (RIG-I)-like RNA helicases (RLRs) and Toll-like receptors (TLRs) (Li et al., 2005). However, the nucleotide-binding domain and leucine-rich repeat (NLR) proteins play an extensive role in the innate immune and inflammatory reactions, and have not been well-studied in the liver and hepatitis virus infection (Qu and Lemon, 2010). RIG-I, melanoma differentiation- associated gene 5 (MDA5) and Toll-like receptor 3 (TLR3) are pathogen-associated pattern recognition receptors, which identify the presence of RNA viruses and stimulate signaling pathways that lead to induction of an antiviral state (Meylan and Tschopp, 2006). RIG-I seems to be the major recognition receptor that recognizes the 5′-triphosphate group (5′-ppp) and blunt end of short (low molecular weight) RNAs with high affinity (Hornung et al., 2006; Pichlmair et al., 2006; Jiang et al., 2011; Luo et al., 2011). In contrast, MDA5 appears to be the major recognition receptor that senses the internal duplex structure of long (high-molecular-weight) double-stranded (ds)RNAs with a weaker affinity (Takeuchi and Akira, 2010; Kato et al., 2011). LGP2 (Laboratory of Genetics and Physiology 2), a cytoplasmic DExH helicase that shares the domain structure of RIG-I and MDA-5 with the exception of the caspase-recruitment domain (CARD), has been reported to exert both positive and negative effects on RIG-I and MDA5 regulation in different cell types in response to different viruses (Venkataraman et al., 2007; Satoh et al., 2010; Kato et al., 2011; Childs et al., 2012; Malur et al., 2012). TLR3 contains an extracellular leucine-rich repeat (LRR) motif, a transmembrane (TM) domain and an intracellular Toll and IL-1R (TIR) domain (Bell et al., 2003; Leulier and Lemaitre, 2008). Compared with short dsRNAs, long dsRNAs are more potent inducers of TLR3 signaling (Bouteiller et al., 2005; Okahira et al., 2005; Leonard et al., 2008; Liu et al., 2008; Pirher et al., 2008; Botos et al., 2009). Currently known RNA-sensing pathways are summarized in Figure 1. MDA5 and TLR3 is likely to sense the genomic RNA of HAV. As the HAV genome possesses the covalently linked 5′ VPg, it is not likely to be sensed by RIG-I, which generally recognizes RNAs with free 5′ triphosphate (Qu and Lemon, 2010). The function of LGP2 during HAV infection was not investigated in details. Changes in the intrahepatic transcriptome during acute HAV infection in experimentally infected chimpanzees were observed by microarray assay. With the exception of CXCL10 (IP10) and interferon-stimulated gene 20 kDa protein (ISG20), both of which are dually regulated by IFN-α/β and IFN-γ, low-level ISG induction is restricted to the first weeks of infection and subsides before peak HAV RNA abundance in the liver (Lanford et al., 2011). The early type I IFN response diminishes before peak replication of HAV within the liver and the onset of liver injury, when a type II IFN (IFN-γ) response becomes evident (Lanford et al., 2011; Zhou et al., 2012; Feng et al., 2015).

Plasmacytoid dendritic cells (pDCs) are “professional” type I IFN-producer cells that play a central role in host antiviral immunity (Gilliet et al., 2008; Reizis et al., 2011). pDCs mainly sense viruses via endosomal TLR7 and TLR9 (Lund et al., 2003), but they can also sense viral nucleic acids in the cytosol (Feng et al., 2015). pDCs require either close contact with cells infected with HAV or exposure to concentrated culture supernatants for IFN-α production. Enveloped virions (eHAV), and not viral RNA exosomes, are responsible for IFN-α induction. Although membrane envelopment protects HAV against neutralizing antibodies, it also facilitates an early but limited detection of HAV infection by pDCs (Feng et al., 2015). During acute hepatitis A (AHA), non-HAV-specific memory CD8^+^ T cells are activated by the IL-15 produced by HAV-infected cells. These CD8^+^ T cells exert innate-like cytotoxicity by activating receptors NKG2D and NKp30 without TCR engagement. Innate-like cytotoxicity of CD8^+^ T cells is associated with liver injury in AHA (Kim et al., 2018).

## HAV evades innate antiviral responses by targeting RLRs/TLRs pathway

HAV was shown to restrain double-stranded RNA (dsRNA)-induced IFNβ gene expression by intervening in RIG-I-mediated IRF3 activation (Brack et al., 2002; Fensterl et al., 2005). Both RIG-I and MDA-5 employ an adaptor protein called MAVS that is localized to the outer mitochondrial membrane via a C-terminal transmembrane domain (Seth et al., 2005). Once activation by RIG-I or MDA-5, MAVS recruits and activates TANK-binding kinase 1 (TBK1) and NF-κB kinase ε (IKKε). TBK1 and IKKε are both accountable for the phosphorylation of IFN regulatory factor 3 (IRF-3), eventually causing IRF-3 dimerization, nuclear translocation, and induction of IFNβ transcription.

HAV proteins 3ABC^pro^ and 2B have been described to interfere with MAVS, thereby disturbing the innate cellular antiviral defense mechanism (Yang et al., 2007; Paulmann et al., 2008). The 3ABC cleavage of MAVS requires both the protease activity of 3C^pro^ and a transmembrane domain in 3A that targets 3ABC to the mitochondria (Yang et al., 2007). The non-structural HAV 2B protein partially colocalizes with MAVS and interferes with the activities of MAVS and the TBK1/IKKε kinases (Paulmann et al., 2008). However, the exact mechanism of this protein still needs to be investigated in detail.

The TLR3 signaling pathway is mediated exclusively by the TRIF adapter, which is recruited to TLR3 by the interaction between the TIR domains of the two molecules (Oshiumi et al., 2003; Yamamoto et al., 2003). TRIF is proteolytically cleaved by 3CD but not by the mature 3C^pro^ protease or the 3ABC precursor that degrades MAVS. 3CD-mediated degradation of TRIF depends on both the cysteine protease activity of 3C^pro^ and the downstream 3D^pol^ sequence but not 3D^pol^ polymerase activity (Qu et al., 2011).

NEMO has been shown to link the TLR3-IRF3 pathway (Zhao et al., 2007). 3C^pro^-mediated proteolytic cleavage of NEMO is directly involved in inhibition of IFN-β transcription (Wang et al., 2014).

**Figure 2:**
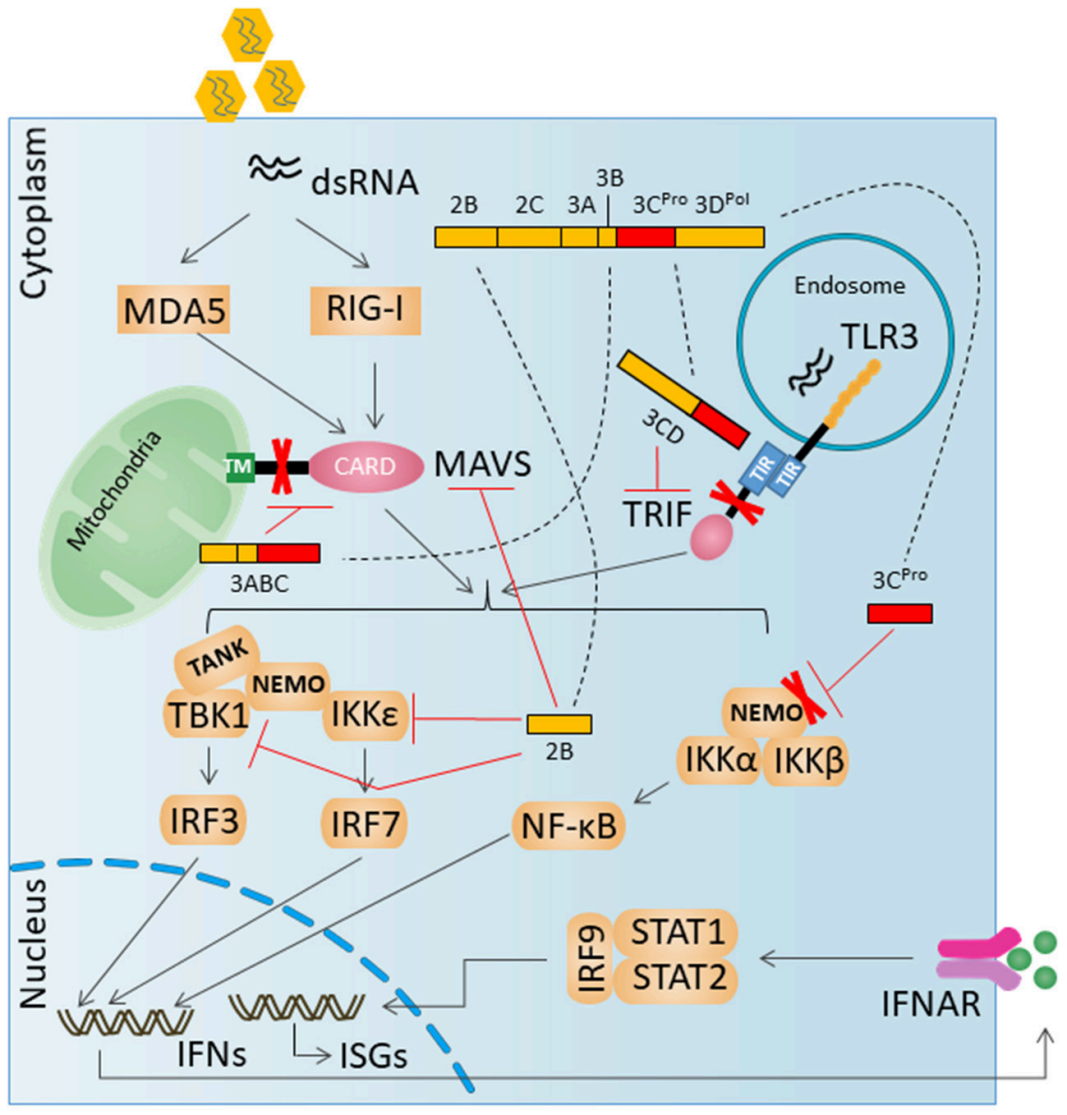
HAV evades innate antiviral responses by degradation key innate immune signaling proteins. A virally encoded, catalytically active polyprotein processing intermediate, 3ABC, degrades MAVS. A second catalytically active 3C^pro^ processing intermediate, 3CD, cleaves the TLR3 adaptor protein TRIF. 3C^pro^ can mediate the degradation of NEMO.

The key innate immune signaling proteins degraded by HAV proteases are illustrated in Figure 2.

## NLR proteins in HAV infection

Nod-like receptors (NLRs) can play an important role in the host response to infections by RNA viruses by both promoting and suppressing innate immunity and inflammation (Wen et al., 2013; Jha and Pan-Yun Ting, 2015). Of note, activated RIG-I has been shown to associate with apoptosis-associated speck-like protein containing CARD (ASC) to form an NLR-independent RIG-I inflammasome complex that induces caspase-1 activation, leading to IL-1β and IL-18 release (Poeck et al., 2010; Pothlichet et al., 2013). Kupffer cells and monocytes express high levels of NLRs, which play important roles in mediating inflammatory responses and modulating liver injury (Dixon et al., 2013). Exposure to either eHAV or HAV neither initiates nor blocks NLRP3 inflammasome assembly or IL-1β secretion by THP-1 cells, a human monocyte cell line derived from an acute monocytic leukemia (Feng and Lemon, 2018). Nonetheless, NLRX1 positively regulates very early (3 h) RLR-induced cytokine responses to HAV in PH5CH8 cells, which are T antigen-transformed adult human hepatocytes. NLRX1 promotes IL-6 and other early cytokine responses by inhibiting activation of the dsRNA-induced PKR. Suppression of PKR activation allows for early, virus-induced increases in synthesis of the IRF1 protein, which plays a key role in regulating these cytokine responses in hepatocytes (Feng et al., 2017).

## The role of innate immunity in host range restriction

The host range of HAV is believed to be restricted to humans and non-human primates. The range of the HAV host species mainly depends on its capacity to evade MAVS- mediated type I IFN responses, which have revealed an unexpected role for MAVS signaling in virus-mediated liver injury (Hirai-Yuki et al., 2016). Type I IFNs, but not type II IFNs, are a major barrier for cross-species infection by HAV. MAVS-dependent, RLR-induced IFN responses play a much more important role in restricting HAV replication than TLR3 *in vivo*, at least in mice, despite the fact that HAV targets adaptors in both signaling pathways for degradation (Feng and Lemon, 2018). Since the sequences targeted in human MAVS and TRIF are not conserved in small mammals, the failure of HAV to infect these species could derive from inability to disrupt IFN responses (Hirai-Yuki et al., 2016). RLR signaling through MAVS in the mitochondria and mitochondria-associated membranes (MAM) results in the expression of both type I and type III interferons, whereas RLR signaling through MAVS in the peroxisome induces the expression of type III interferon alone (Odendall et al., 2014; Bender et al., 2015; Chow et al., 2018). Although type I IFNs clearly play a pivotal role in restricting HAV infection in mice (Hirai-Yuki et al., 2016), type III IFNs are predominantly detected in cultured human hepatocytes infected with HAV (Feng and Lemon, 2018). Human hepatocytes produce and respond robustly to type III interferon as noted above, but mouse hepatocytes express negligible amounts of the receptor for type III IFN and respond poorly to type III interferon (Hermant et al., 2014; Chow et al., 2018). Studies are needed in Ifnlr1^−/−^ mice lacking functional expression of the type III IFN receptor.

## Antiviral response of DExD/H-Box helicases other than the RLRs

Some DExD/H-Box Helicases may act as bona fide RNA sensors, while others may instead act as accessory factors required to promote innate immune signaling through one of the aforementioned RNA-sensing pathways (Figure 2). DDX3 has been shown to bind poly(I:C), VSV RNA, and abortive RNA products from HIV replication (Gringhuis et al., 2017). DHX9 was proposed as an RNA sensor during viral infection in myeloid dendritic cells (Zhang et al., 2011b). A cytosolic complex formed by DDX1, DDX21, and DHX36 has been reported to sense dsRNA in cDCs in order to induce interferons in response to poly(I:C), IAV, and reovirus (Zhang et al., 2011a). DHX33 may act as a sensor for cytosolic PAMP RNA and activate the NLRP3 inflammasome (Mitoma et al., 2013). DHX15 may diverge in its signaling of innate immune responses in different cell types (Lu et al., 2014; Mosallanejad et al., 2014; Wang et al., 2015a). DDX60 has been shown to be required for RIG-I- mediated signaling in response to dsRNA or virus infection (Miyashita et al., 2011; Oshiumi et al., 2015). Small nuclear ribonucleoprotein U5 subunit 200 protein (SNRNP200), typically involved in spliceosome processes, associates with TBK1 to activate IRF3 during SeV infection (Tremblay et al., 2017). The interaction between HAV and these DExD/H-Box Helicases needs to be investigated in detail in future research.

## Antiviral response of other RNA-binding proteins

When activated by binding to dsRNA, oligoadenylate synthetase (OAS) catalyzes the conversion of ATP into 2′-5′-linked oligoadenylates (2–5A), which in turn become second messengers that bind to and activate RNase L. RNase L functions to cleave ssRNA, and the products of this cleavage can cooperate to trigger RIG-I- and MDA5-dependent interferon induction (Malathi et al., 2007, 2010). Protein kinase R (PKR) functions to suppress translation in virus-infected cells by inhibiting eukaryotic translation initiation factor 2A (eIF2A) (Hull and Bevilacqua, 2016). The interferon-induced protein with tetratricopeptide repeats (IFIT) family of proteins can inhibit viral translation and sequester viral RNA (Fensterl and Sen, 2015). LRRFIP1 activates β-catenin to enhance transcriptional activation of Ifnb1 (Yang et al., 2010). A DNA-dependent activator of IRFs (DAI), otherwise known as ZBP1/DLM-1, can activate RIPK1/3/ MLKL (Thapa et al., 2016) and the NLRP3 inflammasome (Kuriakose et al., 2016). High-mobility-group box (HMGB) proteins have been implicated in the signaling of interferon and proinflammatory responses to control virus infection (Ugrinova and Pasheva, 2017). The interplay between some RNA-binding proteins and RLRs pathways is shown in Figure 2. The effect of the aforementioned RNA-binding proteins on HAV hasn't been studied systematically. It would be intriguing to further investigate the function of these RNA-binding proteins during HAV infection.

## Conclusions and perspectives

In HAV-infected cells, viral dsRNA replication intermediates are sensed by cytosolic RLRs (RIG-I and MDA5) as well as endosomal TLR3. However, adaptor proteins MAVS and TRIF and the IκB kinase complex's regulatory subunit, NEMO, are degraded by viral proteinases. This disrupts signals extending from RLRs and TLR3 such that little or no activated IRF3 and NF-κB reach responsive promoters in the nucleus, and therefore, little or no IFNs are produced. Despite many interesting features, HAV remains a largely understudied virus on a fundamental biological level. Future studies on the interactions between HAV and the host immunological system will shed light on the pathogenesis and therapeutic approaches of this virus.

## Author contributions

XC, YX, and JD wrote the review. QX, XY, YZ, BW, HW, JL, KC, and ZM participated in the conception and design of the review. All authors read and approved the final manuscript.

### Conflict of interest statement

The authors declare that the research was conducted in the absence of any commercial or financial relationships that could be construed as a potential conflict of interest.

The handling Editor declared a shared affiliation, though no other collaboration, with several of the authors XC and YZ.
